# Changes in the Systemic Expression of Sirtuin-1 and Oxidative Stress after Intravitreal Anti-Vascular Endothelial Growth Factor in Patients with Retinal Vein Occlusion

**DOI:** 10.3390/biom10101414

**Published:** 2020-10-06

**Authors:** De-Kuang Hwang, Yuh-Lih Chang, Tai-Chi Lin, Chi-Hsien Peng, Ke-Hung Chien, Ching-Yao Tsai, Shih-Jen Chen, Kuan-Hsuan Chen, Min-Yen Hsu

**Affiliations:** 1 Department of Ophthalmology, Taipei Veterans General Hospital, Taipei 112, Taiwan; m95gbk@gmail.com (D.-K.H.); taichilin@hotmail.com (T.-C.L.); Sjchen96@gmail.com (S.-J.C.); 2 School of Medicine, National Yang-Ming University, Taipei 112, Taiwan; 3 Institute of Pharmacology, National Yang-Ming University, Taipei 112, Taiwan; ylchang@vghtpe.gov.tw; 4 Department of Pharmacy, Taipei, Veterans General Hospital, Taipei 112, Taiwan; 5 Institute of Clinical Medicine, National Yang-Ming University, Taipei 112, Taiwan; yred8530@gmail.com; 6 Department of Ophthalmology, Shin Kong Wu Ho-Su Memorial Hospital & Fu-Jen Catholic University, Taipei 24352, Taiwan; chpeng1008@gmail.com; 7 Department of Ophthalmology, Tri-Service General Hospital & National Defense Medical Center, Taipei 114, Taiwan; 8 Department of Ophthalmology, Taipei City Hospital, Taipei 103, Taiwan; tsaikimo@gmail.com; 9 Faculty of Pharmacy, School of Pharmaceutical Sciences, National Yang-Ming University, Taipei 112, Taiwan; 10 Department of Ophthalmology, Chung Shan Medical University Hospital, Taichung 402, Taiwan; 11 School of Medicine, Chung Shan Medical University, Taichung 402, Taiwan; 12 Biotechnology Center, National Chung Hsing University, Taichung 402, Taiwan

**Keywords:** RVO, oxidative stress, SIRT1, vascular endothelial growth factor, VEGF

## Abstract

Objectives: Retinal vein occlusions (RVO) are associated with systemic risk factors. However, the ocular occlusive events might also influence a patient’s systemic condition. This study tried to investigate serum biomarkers associated with oxidative stress, before and after intravitreal anti-vascular endothelial growth factor (aVEGF) therapy in patients with RVOs. Methods: Newly-onset RVO patients were categorized into two groups: comorbid with macular edema requiring aVEGF therapy (treatment group) and no edema (observation group). Age and sex-matched patients (who received cataract surgery) were included as the control group. Intravitreal ranibizumab with a pro-re-nata regimen were administered. Serum samples were collected prior to treatment, at 6 and 12 months after therapy/observation and were collected once before controls who received cataract surgery. mRNA expression of sirtuin-1, its downstream genes, anti-oxidative biomarkers, and proinflammatory cytokines were measured. Results: There were 32, 26, and 34 patients enrolled in the treatment, observation, and control groups, respectively. The expressions of sirtuin-1 and its downstream genes were significantly lower in patients with RVO compared with the control group. Sirtuin-1 gene expression increased after 1 year of aVEGF therapy in the treatment group but remained unchanged in the observation group. Biomarkers of oxidative stress and proinflammatory cytokines were reduced after 1 year of aVEGF therapy. These biomarkers remained with no changes in the observation group. Conclusions: Our study showed that the systemic oxidative stress increased in RVO patients. The aVEGF therapy could alter the gene expression of anti-oxidative proteins and reduce systemic oxidative stress in these patients.

## 1. Introduction

Retinal vein occlusion (RVO) is one of the most common types of retinal disease, and it can lead to devastating vision loss [1]. RVO affects approximately 16 million people around the world and has a 1% to 2% prevalence in people older than 40 years of age [2]. Older age, diabetes mellitus, hypertension, hyperlipidemia, cardiovascular disease, coagulopathy, and glaucoma are known risk factors for RVO. Macular edema (ME) can occur in patients with RVO, leading to severe visual impairment. Various treatments, including laser photocoagulation, surgery, and intravitreal administration of drugs, have been used in the management of ME secondary to RVO. Intravitreal injection of various anti-vascular endothelial growth factors (aVEGFs) is now a widely used treatment for ME and other angiogenic complications of RVO.

In 2015, Brzović-Šarić et al. reported that oxidative stress markers were highly correlative in both the vitreous and serum of patients with proliferative retinopathy compared with patients without metabolic disorders [3]. One of the reasons for this is that many primary retinal cells, such as ganglion cells, would die in the non-nutrition and ischemic conditions that follow vein occlusions, thus leading to an elevation of oxidative stress in the tissue.

Various biomarkers and proteins have been analyzed to help doctors monitor a patient’s level of oxidative stress. Sirtuin-1 (SIRT1) is a nicotinamide adenine dinucleotide-dependent histone deacetylase. Previous studies have demonstrated its role in regulating angiogenesis, as well as its anti-inflammatory and anti-oxidative actions [4,5,6]. Anti-apoptosis, cell cycle regulation, and transcription regulation were included among its many biological functions. Gene expression of SIRT1 can be inhibited by oxidative stress, while conversely, inflammatory responses can be induced by decreasing the activity of SIRT1. Many studies have shown that the level of intraocular oxidative stress would increase in patients with RVO [7]. Our previous work also found that the systemic oxidative status including the gene expression of SIRT1 in patients with RVO is different from normal people who only have cataracts [8]. Analyzing the expression of SIRT using other molecules, such as microRNA-34a (miR-34a) or forkhead box protein O (FOXO) families, may help us to understand the condition of oxidative stress.

Inflammation also plays an important role in RVO patients [9]. When large areas of retinal ischemia occur in RVO, the blood retinal barrier may be impaired and lose its integrity. Proinflammatory cytokines and oxidative stress species may then be systemically influenced [10,11]. Intravitreal aVEGF therapy has been shown to be effective for treating ME in RVO patients; it can restore patients’ vision and prevent the progression of retinal non-perfusion.

In the current study, we aimed to understand the changes in systemic oxidative stress and inflammatory conditions in RVO patients before and after aVEGF therapy. To the best of our knowledge, this is the first study to explore the correlation between ocular treatment of RVO and systemic changes in the expression of SIRT1 and its associated biomarkers.

## 2. Materials and Methods

The present prospective observational study was conducted in accordance with the Declaration of Helsinki. All protocols were approved by the Institutional Review Board of the Taipei Veterans General Hospital (approval nos. 2013-11-012B). All patients provided written informed consent before enrolling in the study.

### 2.1. Patients

Patients who suffered from newly-onset central or branch RVO confirmed by fluorescence angiography in the Taipei Veterans General Hospital were evaluated. Patients were excluded if they had any neurological or cardiac conditions, or any other ocular complication related to RVO. Patients with ME confirmed by optical coherence tomography were categorized into the treatment group and received three monthly intravitreal injections of ranibizumab 0.5 mg, followed by a pro-re-nata (PRN) injection if the central foveal thickness was more than 300 µm. Patients with RVO without ME were followed without any treatment including laser and were categorized as the observation group. Patients were excluded if they did not suffer from ME at enrolment, but it developed during the follow-up period. During the same study period, age- and sex-matched patients without a remarkable ocular or systemic history who underwent cataract surgery were randomly selected and enrolled as the control group.

Blood samples were collected at enrolment from all patients (prior to any treatment) using 5 mL BD K2-ethylenediamine tetraacetic acid (EDTA) tubes. Parts of the patients’ whole blood samples were centrifuged at 2500× *g* at 4 °C for 10 min and then the serum plasma was collected. All mRNAs, miRNAs, cytokines, and enzymes were isolated or extracted from the serum. Additional samples were collected at 6 and 12 months after diagnosis in the treatment and observation groups.

### 2.2. Isolation of mRNA and Quantitative Real-Time Polymerase Chain Reaction (PCR)

Serum RNA was isolated using a RNeasy Plus mini kit (Qiagen, Hilden, Germany). A homology search within the human genome (BLAST, National Center for Biotechnology Information, Bethesda, MD, USA) determined oligonucleotide specificity and dissociation curve analysis was used to confirm it. The quality of RNA was confirmed by an Experion Automated Electrophoresis Station (Bio Rad, Hercules, CA, USA). The sequences of all tested genes are shown in Table 1. Oligonucleotides for SIRT1, peroxisome proliferator-activated receptor gamma (PPAR-r), peroxisome proliferator-activated receptor gamma coactivator 1-alpha (PGC-1α), FOXO-1, FOXO-3, and β-actin were designed using the computer software package Primer Express 2.0 (Applied Biosystems, Foster City, CA, USA) and they were used to measure the corresponding gene expression. Invitrogen (Breda, The Netherlands) synthesized all oligonucleotides. PCR was performed using SYBR Green with an ABI 7000 sequence detection system (Applied Biosystems) according to the manufacturer’s guidelines.

The blood micro-RNAs (miRNA) were isolated using a PAXgene blood RNA kit. Primers specific for miR-34a were obtained from Applied Biosystems (Foster City, CA, USA) to execute quantitative real-time PCR according to the vendor’s protocol for TaqMan miRNA assays. miRNA was investigated using a PAXgene blood mRNA kit with the 1/ΔCT method. U6 was used as the control to calculate the relative expression levels of miR-34a.

### 2.3. Measuring Antioxidant Enzyme Activity, Hydrogen Peroxide (H_2_O_2_), and Cytokines

Enzyme-linked immunosorbent assays (ELISAs) were performed using commercial kits according to the manufacturer’s instructions, to determine superoxide dismutase (SOD; Cell Biolabs, San Diego, CA, USA, STA-340), catalase (Cell Biolabs, STA-341), and H_2_O_2_ (Cell Biolabs, STA-344) levels in the plasma. The enzyme activities of SOD and catalase were reported as units per milligram of protein. The concentration of H_2_O_2_ was reported in µM. The concentration of various pro-inflammatory markers, including interleukin (IL)-6, IL-8, and tumor necrosis factor (TNF)-α were also measured and reported in pg/mL.

### 2.4. Statistical Analysis

All results were reported as the mean ± standard deviation. Variables were compared using one-way analysis of variance (ANOVA) with Bonferroni’s correction, Wilcoxon signed-rank test or Pearson’s chi-squared test, as appropriate. The correlation between changes in gene expression and patients’ visual acuity was also analyzed. Statistical analysis was performed using SPSS version 13.0 (SPSS, Inc., Chicago, IL, USA). Statistical significance was set at *p* <0.05.

## 3. Results

There were 32 patients diagnosed as having RVO and secondary ME who underwent aVEGF therapy and were enrolled in the treatment group. Of these patients, 20 were diagnosed with branch RVO (BRVO, median age = 78.4 years), 7 with ischemic central RVO (CRVO, median age = 77.3 years), and 5 with non-ischemic CRVO (median age = 76.2 years). A total of 26 RVO patients (median age = 70.3) who never developed ME were enrolled in the observation group. A total of 34 age- and sex-matched cataract patients were randomly selected into the control group (median age = 73.1 years). Basic characteristics of these patients are shown in Table 2.

### 3.1. Intravitreal aVEGF Treatment Was Associated with Increased SIRT1 Gene Expression, Its Downstream Genes, and Other Anti-Oxidative Enzymes in Patients with RVO

**Figure 1:**
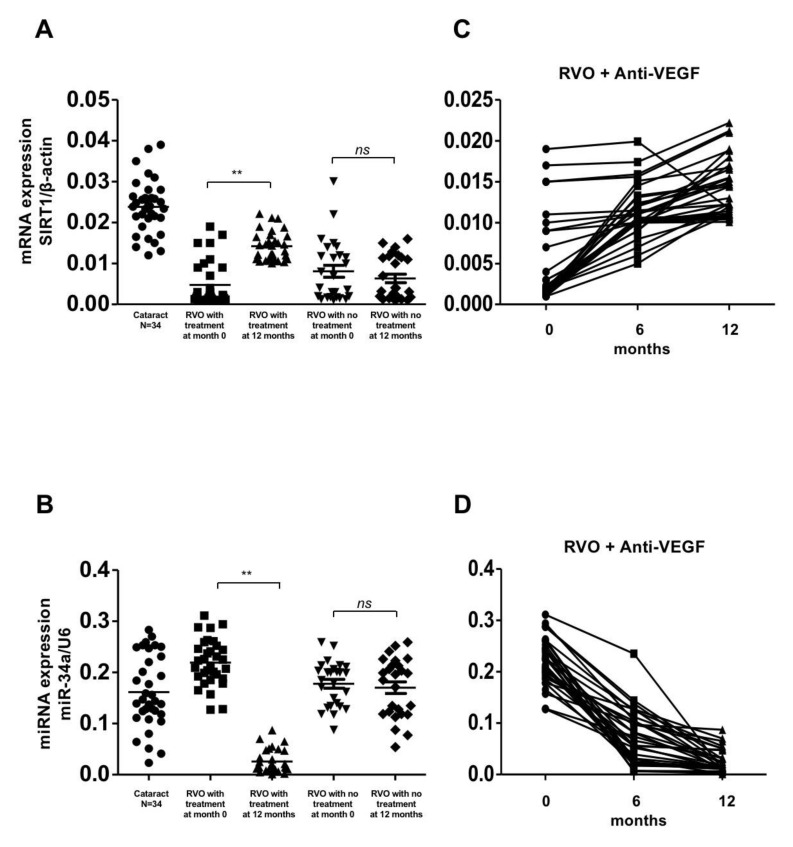
Gene expression of SIRT1 and miR-34a in patients with retinal vein occlusion (RVOs) under anti-vascular endothelial growth factor(aVEGF) therapy, without therapy, and controls. (**A**) Gene expression of SIRT1 were lower in patients with RVOs than controls at baseline (0.005 ± 0.005, 0.008 ± 0.007, and 0.024 ± 0.007, respectively, *p* < 0.05). One year later, the expression increased in patients who received aVEGF therapy (0.014 ± 0.004, ** *p* < 0.01) but remained in patients without therapy (0.006 ± 0.005, ns: difference was not significant). (**B**) Gene expressions of miR-34a were higher in patients with RVOs than controls at baseline (0.219 ± 0.045, 0.178 ± 0.044, and 0.162 ± 0.071, respectively, *p* < 0.05). One year later, the expression decreased in patients who received aVEGF therapy (0.026 ± 0.022, ** *p* < 0.01) but remained in patients without therapy (0.170 ± 0.058, ns: difference was not significant). (**C**) There was a gradually increasing trend of SIRT1 gene expression in RVO patients who received aVEGF therapy 6 months and 12 months after intervention. (**D**) There was a gradually decreasing trend of miR-34a gene expression in RVO patients who received aVEGF therapy 6 months and 12 months after intervention.

**Figure 2:**
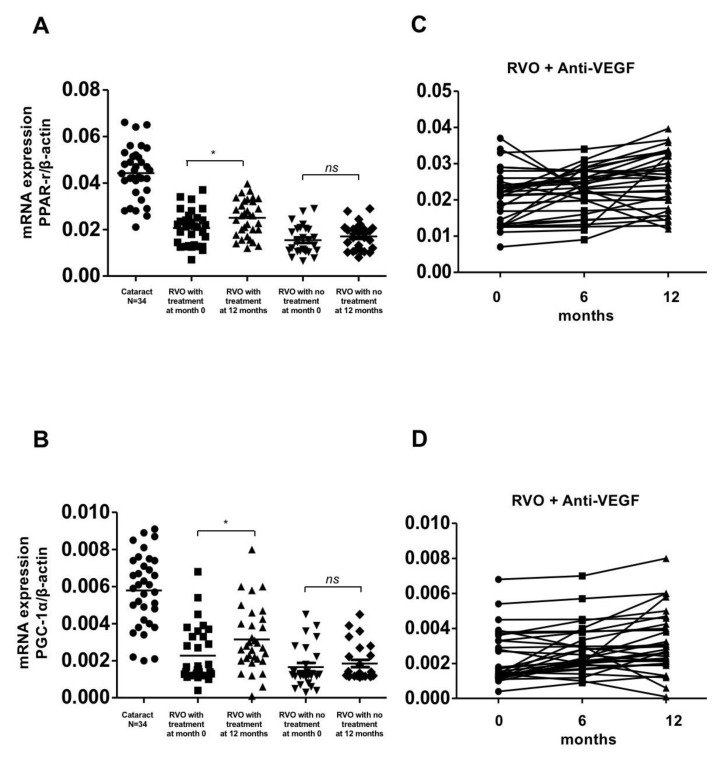
Gene expression of PPAR-r and PGC-1α in patients with retinal vein occlusion (RVOs) under anti-vascular endothelial growth factor (aVEGF) therapy, without therapy, and controls. (**A**) Gene expression of PPAR-r were lower in patients with RVOs than controls at baseline (0.020 ± 0.007, 0.015 ± 0.006, and 0.044 ± 0.011, respectively, * *p* < 0.05). One year later, the expression increased in patients who received aVEGF therapy (0.025 ± 0.008, * *p* < 0.05) but remained in patients without therapy (0.017 ± 0.006, ns: difference was not significant). (**B**) Gene expression of PGC-1α were lower in patients with RVOs than controls at baseline (0.002 ± 0.001, 0.002 ± 0.001, and 0.006 ± 0.002, respectively, * *p* < 0.05). One year later, the expression increased in patients who received aVEGF therapy (0.003 ± 0.002, * *p* < 0.05) but remained in patients without therapy (0.002 ± 0.001, ns: difference was not significant). (**C**) There was a gradually increasing trend of PPAR-r gene expression in RVO patients who received aVEGF therapy 6 months and 12 months after intervention. (**D**) There was a gradually increasing trend of PGC-1α gene expression in RVO patients who received aVEGF therapy 6 months and 12 months after intervention.

**Figure 3:**
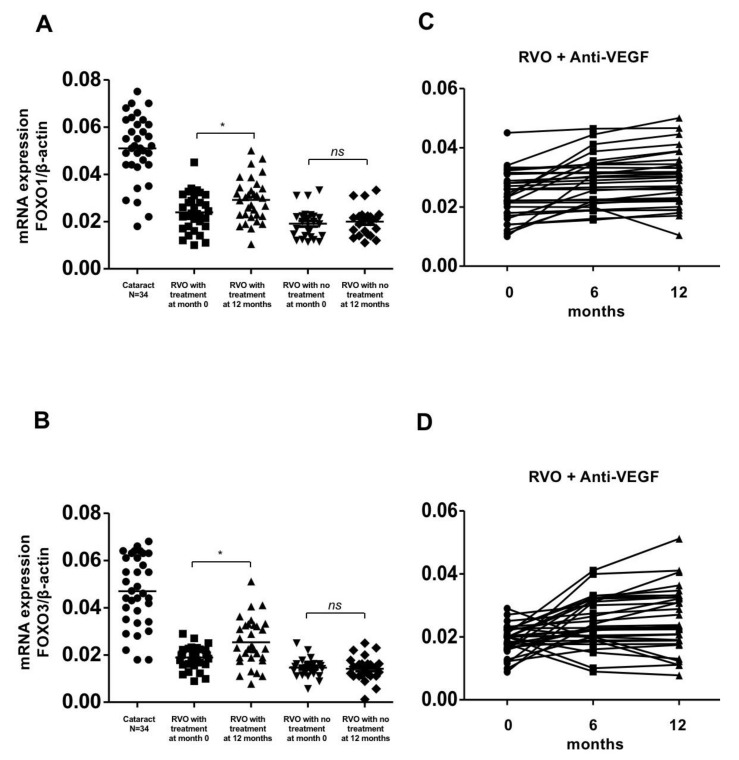
Gene expression of FOXO-1 and FOXO-3 in patients with retinal vein occlusion (RVOs) under anti-vascular endothelial growth factor (aVEGF) therapy, without therapy, and controls. (**A**) Gene expression of FOXO-1 were lower in patients with RVOs than controls at baseline (0.024 ± 0.008, 0.002 ± 0.006 and 0.051 ± 0.014, respectively, * *p* < 0.05). One year later, the expression increased in patients who received aVEGF therapy (0.029 ± 0.009, * *p* < 0.05) but remained in patients without therapy (0.020 ± 0.006, ns: difference was not significant). (**B**) Gene expression of FOXO-3 were lower in patients with RVOs than controls at baseline (0.020 ± 0.005, 0.015 ± 0.004, and 0.047 ± 0.015, respectively, * *p* < 0.05). One year later, the expression increased in patients who received aVEGF therapy (0.025 ± 0.010, * *p* < 0.05) but remained in patients without therapy (0.014 ± 0.005, ns: difference was not significant). (**C**) There was a gradually increasing trend of FOXO-1 gene expression in RVO patients who received aVEGF therapy 6 months and 12 months after intervention. (**D**) There was a gradually increasing trend of FOXO-3 gene expression in RVO patients who received aVEGF therapy 6 months and 12 months after intervention.

The expression of SIRT1, PPAR-r, PGC-1α, FOXO-1, and FOXO-3 were significantly lower in the treatment and observation groups compared with the control group at initial presentation (*p* < 0.01). The expression of these genes significantly increased in the treatment group after 1 year of aVEGF therapy (0.005 ± 0.005 to 0.014 ± 0.004, 0.02 ± 0.007 to 0.025 ± 0.008, 0.002 ± 0.001 to 0.003 ± 0.002, 0.024 ± 0.008 to 0.029 ± 0.009, and 0.02 ± 0.005 to 0.025 ± 0.010, respectively, with *p*-values of <0.01, <0.05, <0.05, <0.05, and <0.05, respectively). There was a gradual upward trend in the expression of these genes during the follow up period. Conversely, the expression of these anti-oxidative enzymes did not show any significant changes in those patients who had never had ME and received aVEGF therapy (Figure 1A,C, Figure 2 and Figure 3).

### 3.2. miR-34a Expression Was Higher in Patients with RVO and Secondary ME; Intravitreal aVEGF Therapy Was Associated with a Reduction in miR-34a Gene Expression

The gene expression of miR-34a was significantly higher in patients who suffered from RVO and secondary ME. A trend of decreasing expression was observed during follow-up and a significant reduction was identified after 1 year of aVEGF therapy (0.219 ± 0.045 to 0.026 ± 0.022; *p* <0.01; Figure 1B,D). There was no significant difference in miR-34a gene expression between RVO patients without ME and the control group, or between the initial presentation and 1-year follow-up in the observation group (Figure 1B).

### 3.3. Biomarkers and Other Enzyme Activities Changed after aVEGF Therapy in Patients with RVO and Secondary ME

**Figure 4:**
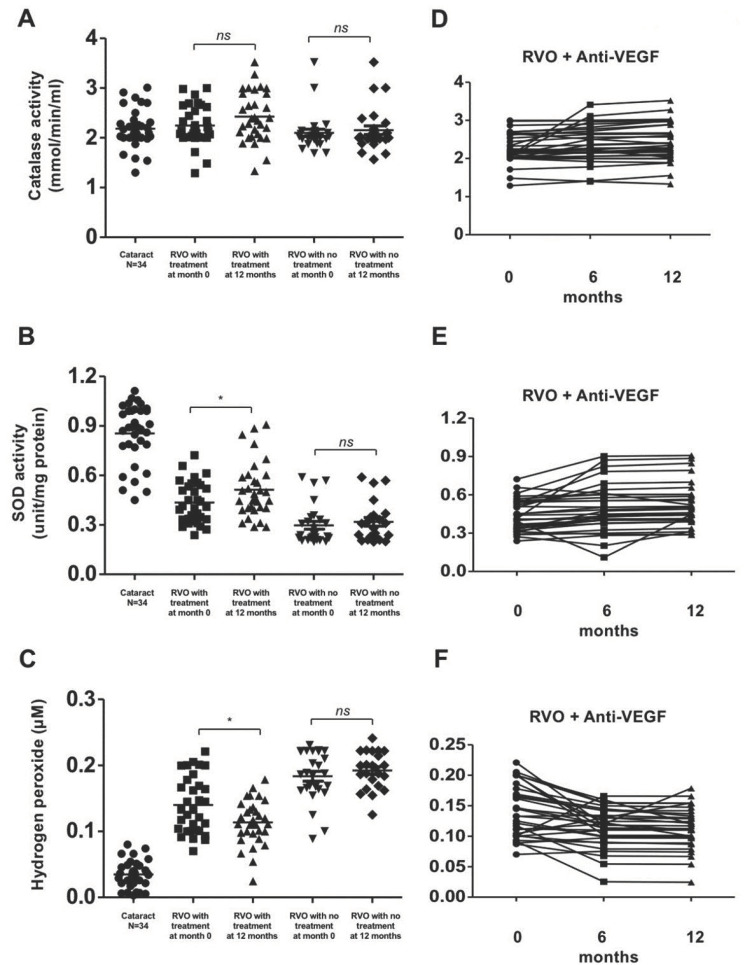
Antioxidant enzyme activity and ROS concentrations in patients with retinal vein occlusion (RVOs) under anti-vascular endothelial growth factor (aVEGF) therapy, without therapy, and controls. (**A**) There was no difference in activity of serum catalase among the treatment, observation, and control group (2.240 ± 0.390, 2.100 ± 0.378, and 2.183 ± 0.387 mmol/min/mL, respectively, ns: difference was not significant) at baseline. One year later, the activity of serum catalase remained unchanged in patients who received aVEGF therapy or not (2.430 ± 0.503 and 2.152 ± 0.441 mmol/min/mL, ns: differences were all nonsignificant). (**B**) The activities of SOD were lower in patients with RVOs than controls at baseline (0.435 ± 0.124, 0.297 ± 0.118, and 0.854 ± 0.181 unit/mg, respectively, * *p* < 0.05). One year later, the activity increased in patients who received aVEGF therapy (0.512 ± 0.168, * *p* < 0.05) but remained in patients without therapy (0.318 ± 0.123, ns: difference was not significant). (**C**) The concentrations of hydrogen peroxide were significantly higher in patients with RVOs than controls at baseline (0.140 ± 0.043, 0.184 ± 0.038, and 0.035 ± 0.020 uM, respectively, * *p* < 0.05). One year later, the concentration decreased in patients who received aVEGF therapy (0.114 ± 0.033uM, * *p* < 0.05) but remained in patients without therapy (0.198 ± 0.028 uM, ns: difference was not significant). (**D**) The activity of serum catalase remained unchanged in RVO patients who received aVEGF therapy 6 months and 12 months after intervention. (**E**) There was a gradually increasing trend of SOD activity in RVO patients who received aVEGF therapy 6 months and 12 months after intervention. (**F**) There was a gradually decreasing trend of hydrogen peroxide concentration in RVO patients who received aVEGF therapy 6 months and 12 months after intervention.

There was no significant difference in the level of catalase activity in all three groups before and after 1 year of treatment/observation (Figure 4A). The concentration of H2O2 was significantly higher in RVO patients compared with the control group (*p* < 0.01). These results showed that the concentration of serum H2O2 significantly decreased in RVO patients with ME after treatment (0.14 ± 0.043 to 0.114 ± 0.033 μM; *p* < 0.05) but remained high in patients who did not receive aVEGF therapy (Figure 4C).

Conversely, SOD concentration was significantly lower in patients with RVO than control group. It significantly increased after 1 year of aVEGF therapy in the treatment group (0.435 ± 0.124 to 0.512 ± 0.168 unit/mg; *p* < 0.05). The concentration of SOD remained low in RVO patients who did not receive aVEGF therapy (Figure 4B).

### 3.4. Systemic Concentration of Pro-Inflammatory Cytokines Decreased after aVEGF Therapy

**Figure 5:**
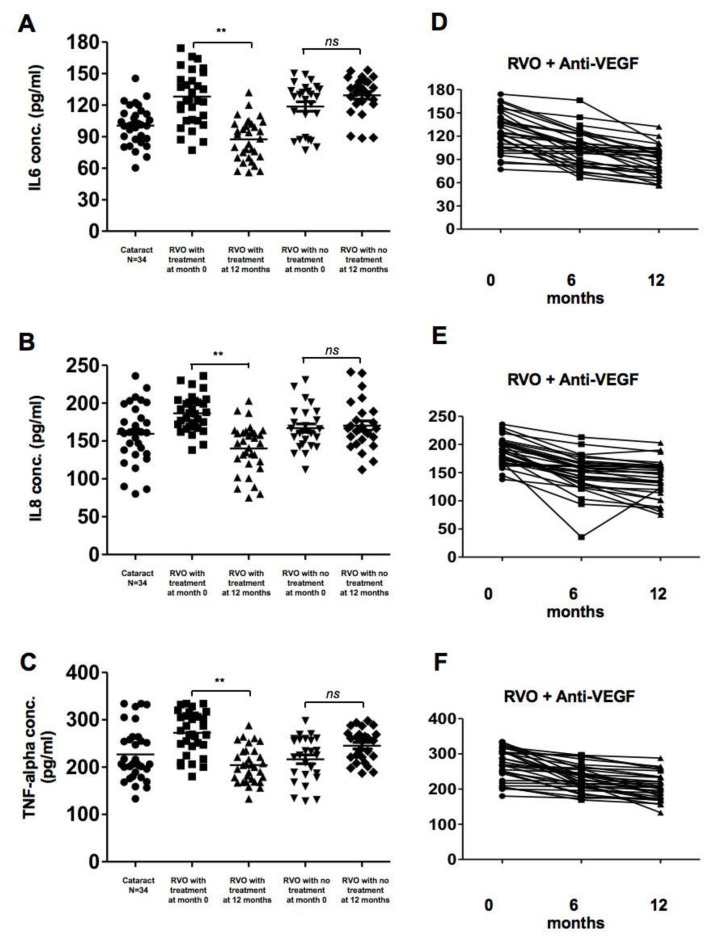
Concentrations of pro-inflammatory cytokines in patients with retinal vein occlusion (RVOs) under anti-vascular endothelial growth factor (aVEGF) therapy, without therapy, and controls. (**A**) The concentrations of interleukin (IL)-6 were significantly higher in patients with RVOs than controls at baseline (128.0 ± 27.3, 118.6 ± 23.3, and 100.5 ± 18.25 pg/mL, respectively, *p* < 0.05). One year later, the concentration decreased in patients who received aVEGF therapy (87.31 ± 19.53, ** *p* < 0.01) but remained in patients without therapy (129.2 ± 18.3, ns: difference was not significant). (**B**) The concentrations of IL-8 were higher but not statistically significant in patients with RVO an macular edema than those without macular edema and controls at baseline (186.6 ± 23.6, 167.0 ± 27.9, and 159.5 ± 38.1 pg/mL, respectively, ns: difference was not significant). One year later, the concentration decreased in patients who received aVEGF therapy (140.2 ± 32.0 pg/mL, ** *p* < 0.01) but remained in patients without therapy (170.3 ± 32.0 pg/mL, ns: difference was not significant). (**C**) The concentrations of Tumor Necrosis Factor-alpha were significantly higher in patients with RVO an macular edema than those without macular edema and controls at baseline (272.4 ± 45.0, 216.4 ± 47.3, and 226.7 ± 55.5 pg/mL, respectively, *p* < 0.05). One year later, the concentration decreased in patients who received aVEGF therapy (204.3 ± 37.75 pg/mL, ** *p* < 0.01) but remained in patients without therapy (245.3 ± 34.4 pg/mL, ns: difference was not significant). (**D**) There was a gradually decreasing trend of IL-6 concentration in RVO patients who received aVEGF therapy 6 months and 12 months after intervention. (**E**) There was a gradually decreasing trend of IL-8 concentration in RVO patients who received aVEGF therapy 6 months and 12 months after intervention. (**F**) There was a gradually decreasing trend of Tumor Necrosis Factor-alpha concentration in RVO patients who received aVEGF therapy 6 months and 12 months after intervention.

The concentration of IL6, IL8, and TNF-α in the serum was measured using commercial ELISA kits. A significant decrease in these cytokines was observed in the treatment group after 1 year of aVEGF therapy (128 ± 27.3 to 87.31 ± 19.53, 186.6 ± 23.6 to 140.2 ± 32.03, and 272.4 ± 44.95 to 204.3 ± 37.75 mg/dL, respectively; *p* < 0.01 in each comparison). These cytokines were not significantly different after 1 year of observation in RVO patients who did not receive aVEGF therapy (Figure 5).

### 3.5. Changes in SIRT1 and miR34a Were Positively Correlated with Visual Acuity in RVO Patients

**Figure 6:**
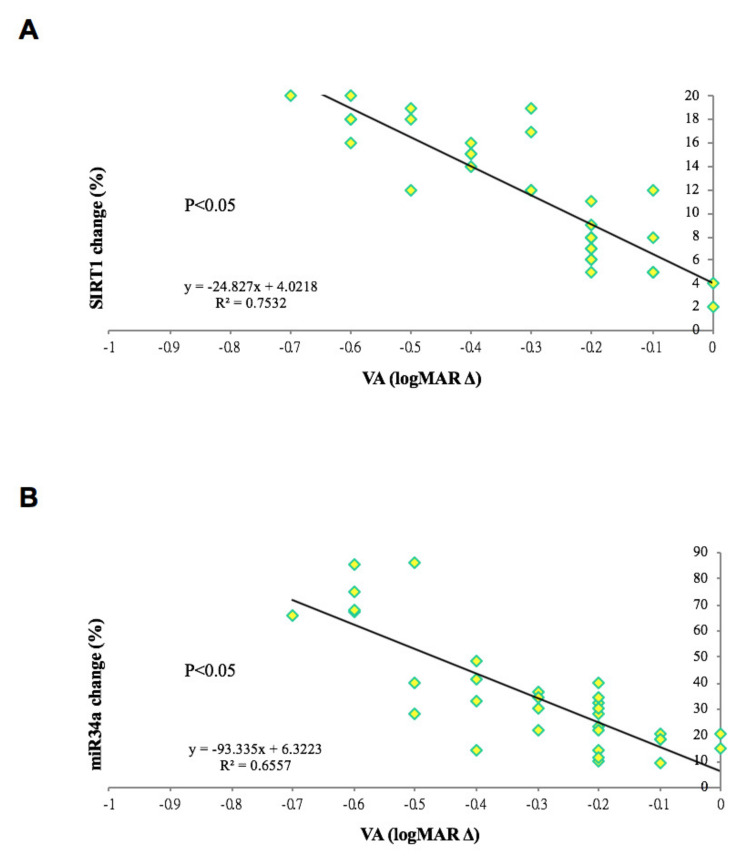
Correlation between changes in gene expression and visual outcome of patients with retinal vein occlusions and macular edema. (**A**) The absolute difference of SIRT1 gene expression (in percentage) was positively correlation to the improvement of visual acuity between base line and 1-year outcome in patients with retinal vein occlusion and secondary macular edema. (R^2^ = 0.7532, *p* < 0.05). (**B**) The absolute difference of miR-34a gene expression (in percentage) was positively correlation to the improvement of visual acuity between base line and 1-year outcome in patients with retinal vein occlusion and secondary macular edema. (R^2^ = 0.6557, *p* < 0.05).

Interestingly, the study data showed that the improvement in visual acuity in patients with RVO and secondary ME after 1 year of aVEGF therapy was positively associated with the absolute difference in SIRT1 and miR-34a expression before and after treatment (R2 = 0.753 and 0.656, respectively; *p* < 0.05; Figure 6).

## 4. Discussion

Systemic oxidative stress has been found to contribute to the pathophysiology of various diseases, including neoplastic, degenerative, and inflammatory diseases. Ocular disease used to be thought of as a localized problem that would not substantially affect the patient’s physical condition. However, the results of the current study revealed that patients with RVO and ME may have increased systemic oxidative stress and inflammation biomarkers, as well as reduced expression of anti-oxidative enzymes in their serum. This condition could be modified and improved by local therapy.

Previous literature has demonstrated that the integrity of the blood-retinal barrier can be disrupted if there is a large area of retinal ischemia and inflammation. It is reasonable to assume that intraocular reactive oxygen species and cytokines may enter the systemic circulation through the impaired blood-retinal barrier in patients with RVO. In the present study, the gene expression of SIRT1 was decreased in the serum of patients with RVO, regardless of whether they had secondary ME. Intravitreal aVEGF therapy attenuated systemic oxidative stress and resulted in increased SIRT1 expression after 1 year. Treatment could further enhance the gene expression of FOXO, PGC-1 and PPAR-r [12,13].

SIRT1 can control reactive oxygen species generation by modulating PGC-1 activity [14,15,16]. Our data are comparable with previous studies, indicating that PGC-1 was up-regulated secondary to SIRT1 activation. FOXO transcription factors act at the interconnections between metabolic pathways and are inducible by many important signal transducers and mediators. In the current study, it was found that FOXO1 and FOXO3 gene expression were up-regulated after intravitreal aVEGF treatment. As for diverse results from other biomarkers of oxidative stress and anti-oxidative enzymes, it was also found that the activities of serum SOD and H_2_O_2_ were altered by RVO and its treatments. In addition, an association between changes in gene expression and clinical responses to treatment were also identified. This could indicate that the more vision patients regained, the less permanent cell damage they suffered and the higher their reduction in systemic oxidative stress.

An association between inflammatory cytokines and RVO has already been proven in many previous articles [9,17]. Clinically, intravitreal corticosteroids are an effective therapy for treating ME secondary to RVO [18,19]. Moreover, SIRT1 is a negative regulator of p53, which also plays a key role in inflammatory diseases; therefore, these inflammatory cytokines could also be found systemically in RVO patients. The results of the current study showed that the systemic concentrations of inflammatory cytokines in RVO patients decreased 1 year after serial aVEGF therapy.

Ranibizumab is a humanized monoclonal aVEGF antibody, which does not contain an Fc section [20,21]. Previous studies have shown that the systemic concentration of this antibody is low and minimally affects systemic cytokines. All treated patients in the present study received ranibizumab monotherapy. In addition, blood samples from patients in the treatment group were collected after more than 4 weeks of intraocular injection. Thus, the authors assumed that the biomarkers of oxidative stress in the patients’ serum were less likely to have been influenced by the intravitreal injection itself [22].

There were several limitations to the present study. First, the gene expression and concentration of the investigated enzymes and biomarkers was not assessed in the eye, which may have given more information about the correlation between the intraocular and systemic molecular environments. Further studies investigating intraocular enzymes should be conducted to confirm the findings and conclusions of the current study. Second, RVOs, especially central RVOs, usually relate to systemic vascular diseases. This study only included patients without a major history of cardiac and systemic diseases. However, no blood or echogram exam were taken/performed to evaluate the patients’ general condition, which may also have influenced the level of systemic oxidative stress. Systemic vascular diseases have been proven relating to oxidative stress. These diseases could not only be triggered by oxidative stress but also altered systemic oxidative status. RVO patients comorbid with these diseases might also show a higher level of oxidative stress, angiogenesis, and inflammatory cytokines in their serum. Treatments for these diseases might also alter systemic oxidative and inflammatory status. Therefore, it cannot be confirmed that the gene expression and biomarker concentrations that were initially recorded in our results were entirely a result of intraocular disease. Fortunately, most of the patients did not receive any systemic treatments other than hypertension, and their treatments were not significantly changed during the study period. Therefore, we can assume that all changes in gene expression and biomarker concentration were modified by intraocular therapy. Third, it is possible that the systemic effects are the result of intravitreally administered anti-VEGF entering the circulation and transiently suppressing the circulating level of VEGF. Besides, we only checked the gene expression of SIRT1 in this study to evaluate the upstream systemic reaction from ocular vascular event. Although we found a correlation between RVO and level of gene expression of SIRT1 in serum, it is important to evaluate the actual pathophysiological influence on the human body. We believed that the changes of SIRT1 protein level in serum and intraocular fluid should be evaluated in patients suffering from RVO in the future work. Lastly, all patients in the treatment group received different doses of intravitreal injections based on their required regimen. Since the severity of vascular occlusions in each patient was also different, it was hard for us to find an exact correlation between intraocular aVEGF injection dose and systemic anti-oxidative change. Nevertheless, an association was identified between the improvement in clinical visual acuity and changes in systemic gene expression. These findings support our theory that the systemic change observed after treatment in the current study might be a result of therapy for ocular disease.

In addition, elevated oxidative stress-related biomarkers were also found in the control group of RVO patients without ME, and these remained during follow-up. We suggested that the changes of systemic inflammatory and oxidative status observed in our study were mainly secondary to the local event. These changes in systemic inflammatory and oxidative status may further influence other organs and contribute to the development or progression of some diseases such as hypertension and degenerative diseases. Findings in this study not only implicated that systemic inflammation and oxidative stress followed by RVO should probably be monitored but also suggested the possible consideration regarding intraocular inflammatory or anti-oxidative therapy during the acute phase of retinal vein occlusive events. Furthermore, our results also indicated that the systemic influence of RVO in these patients should be considered when deciding whether to treat these patients or not.

## 5. Conclusions

**Figure 7:**
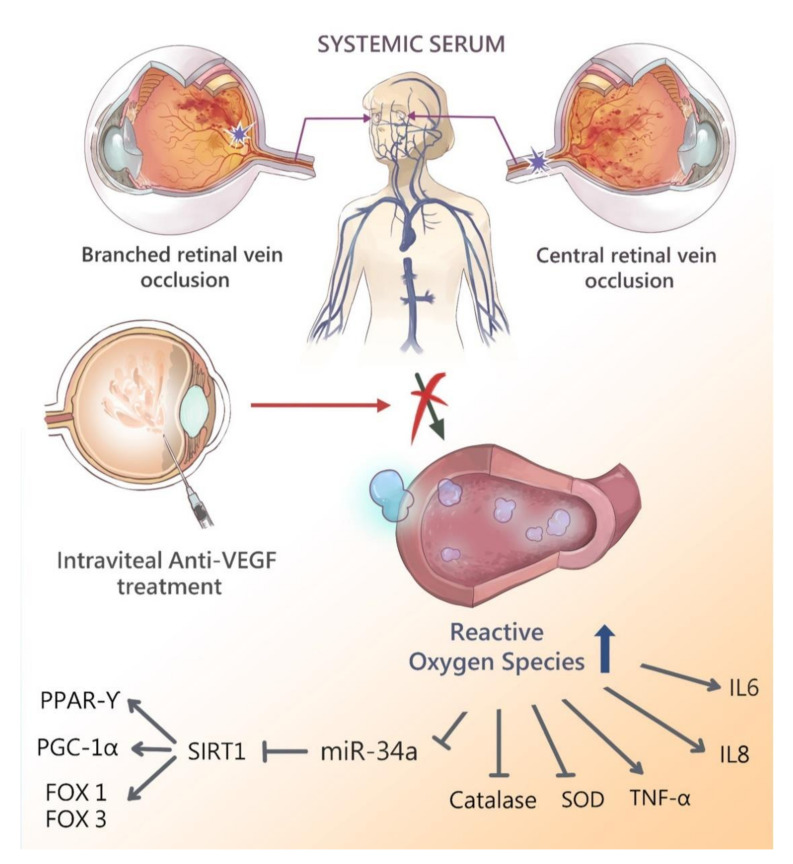
Schematic diagram showing the possible epigenetic regulation in patients with retinal vein occlusions after intravitreal anti-vascular endothelial growth factor therapy. Retinal vein occlusion leads to elevation of reactive oxygen species and release to serum due to impaired blood-retinal-barrier. Antioxidant enzymes such as SIRT1 and SOD are decreased due to the serum oxidative stress scavenge by those enzymes. Downstream antioxidant enzymes PPAR-r, PGC-1α, FOXO1, and FOXO3 are also decreased. Pro-inflammatory cytokine including IL-6, IL-8 and TNF-α are elevated. Intravitreal anti-VEGF treatment reduces the ROS concentration (e.g., H_2_O_2_) and improves activities of various antioxidants enzymes, enhance gene expression of SIRT1 and miR-34a, and subsequently up-regulates PPAR-r, PGC1-α, FOXO1, FOXO3 expression. The cytokine expression of IL-6, IL-8 and TNF-α are decreased after intravitreal anti-vascular endothelial growth factor therapy.

In conclusion, the current study found that intravitreal aVEGF therapy may enhance systemic SIRT1 gene expression and up-regulate its downstream genes (Figure 7). It was demonstrated that after 1 year of therapy, the level of systemic oxidative stress was reduced in patients with RVO and secondary ME. This study indicated the systemic beneficial effects of intravitreal aVEGF therapy in RVO patients.

## Figures and Tables

**Table 1:** Primers for real time PCR.

Gene	Sense	Anti-Sense
*miR-34a*	5′-CGGTATCATTTGGCAGTGTCT-3′	5′-GTGCAGGGTCCGAGGT-3′
*SIRT1*	5′-TGTGGTAGAGCTTGCATTGATCTT-3′	5′-GGCCTGTTGCTCTCCTCAT-3′
*PPAR-r*	5′-AGTGTGAATTACAGCAAATCTCTGTTTT-3′	5′-GCACCATGCTCTGGGTCAA-3′
*PGC-1α*	5′-CCGCACGCACCGAAA-3′	5′-TCGTGCTGATATTCCTCGTAGCT-3′
*FOXO1*	5′-ATGGTCAAGAGCGTGCCC-3′	5′-GATTGAGCATCCACCAAG-3′
*FOXO3*	5′-TCTCCCGTCAGCCAGTCTAT-3′	5′-AGTCACTGGGGAACTTGTCG-3′
*β-actin*	5′-CGGGAAATCGTGCGTGAC-3′	5′-TGCCCAGGAAGGAAGGCT-3′

**Table 2:** Clinical characteristics of study population.

	**Cataract (Reference Values) n = 34**	**Treatment Group (CME Due to RVO Underwent IVI Ranibizumab) n = 32**			**No Treatment Group n = 26**
		BRVO (with CME) n = 20	CRVO (Ischemic) n = 7	CRVO (Non-Ischemic) n = 5	RVO without CME
Age, years	73.1 ± 6.2	78.4 ± 2.6	77.3 ± 3.2	76.2 ± 3.8	70.3 ± 3.4
Female/Male	18/16	11/9	5/2	2/3	15/11
Body mass index, kg/m^2^	23.8 ± 2.2	23.7 ± 2.8	23.9 ± 3.9	24.6 ± 3.2	22.1 ± 3.5
Hypertension	17	13	7	5	10
Diabetes mellitus	0	0	0	0	0
Alzheimer’s disease	0	0	0	0	0
Parkinson disease	0	0	0	0	0
eGFR, mL/min/1.73 m^2^	63.1 ± 3.5	61.5 ± 4.8	63.4 ± 5.6	63.4 ± 5.6	64.2 ± 3.6
ACE inhibitor/ARB use	16	12	6	4	10

Data expressed as mean ± standard deviation. RVO: retina vein occlusion. CME: cystoid. macular edema. IVI: intravitreal injection. eGFR: estimated Glomerular filtration rate. ACE: angiotensin-converting. enzyme. ARB: Angiotensin II receptor blocker.
